# Medicinal Properties of Food

**Published:** 1891-06

**Authors:** 


### MEDICINAL PROPERTIES OF FOODS.

The medicinal properties of some foods are as definite as their nutritive, and as reliable as those of drugs ; indeed, the pharmacopœia includes the following so-called foods in its list of official drugs : asparagus, cabbage, carrots, dandelion, endive, garlic, Indian corn, leeks, lettuce, mushrooms, parsnips, sweet potatoes, sorrel, sea weeds, tomatoes, the cereals, spices, sweet herbs, sugar, honey, molasses, the various berries, the oily nuts, animal fats, eggs, oysters, many kinds of fish, nearly all fruits and their jellies and their juices, the wines, cordials, and spirits, the condiments, and salt especially, without which life is insipid indeed. In their order the dietetic qualities of the condiments will be considered, and the special action of various foods in connection with the various ways of preparing them.

Just as common salt is indispensable to the health of cattle, the salts of potash present in vegetables and fruit are important to man. The good health of many vegetarians is due to this substance.

The cookery of vegetables largely affects their dietetic value, and mistresses should instruct their cooks as to the proper ways of treating these important foods. In the first place, the most strict attention should be paid to cleansing them thoroughly from dust, sand, and injurious worms and insects; careful washing in plenty of cold salted water, with a brush or cloth, will suffice for roots, and careful examination of the leaves of succulent plants; the latter should be left blossom end down in enough salted cold water to cover them for at least an hour. This will generally destroy all parasite life, so that the small creatures will fall of their own weight to the bottom of the water, unless enfolded between the leaves; therefore cabbage, lettuce, etc., should be carefully inspected before cooking. Soft water in cooking will so far soften the tissues of vegetables as to extract much of their juices and valuable salts, unless salt is added to it; and the boiling of peeled vegetables withdraws much of their potash, the value of which we shall presently see. French cooks, having first thoroughly cleansed them, will save the water in which they have been boiled for sauces and soups. We would suggest moderate salting, and then have the water used in making the white sauce usually served with vegetables.

